# Clever pest control? The role of cognition in biological pest regulation

**DOI:** 10.1007/s10071-022-01731-4

**Published:** 2022-12-17

**Authors:** Deyatima Ghosh, Elizabeth A. John, Anna Wilkinson

**Affiliations:** 1 Department of Life Sciences, University of Lincoln, Lincoln, UK; 2 SM Sehgal Foundation Center for Biodiversity and Conservation, Ashoka Trust for Research in Ecology and the Environment, PO, Royal Enclave, Srirampura, Jakkur, Bangalore, 560064 India

**Keywords:** Biological pest control, Pest management, Cognition, Learning, Ecosystem services

## Abstract

Crop pest management is a global challenge. Increases in agricultural intensity due to anthropogenic demands, alongside the need to reduce the reliance on pesticides to minimize environmental harm, have resulted in an urgent need to improve and expand other methods of pest control. One increasingly utilized method is biological pest control, in which natural pest predators are used to regulating crop pests. Current approaches to biological pest regulation assess the importance of a pest controller by examining its ability to maintain pest populations over an extended period. However, this approach lacks efficiency, specificity, and efficacy because it does not take into account crucial factors which determine how predators find, evaluate and remember food sources—the cognitive processes underlying their behavior. This review will investigate the cognitive factors involved in biological pest control and examine how these factors may be manipulated to impact pest behavior and pest controller performance.

## Introduction

The field of animal cognition has blossomed over the last 25 years (see other articles in this Special Issue) and we are gaining a better understanding of cognitive processes in animals. However, in this review, rather than considering what advances have been made in the last 25 years, we want to consider potential directions of the field in the next 25 years. Inevitably, we will continue to diversify the species we study and the contexts in which they are studied and gain a more thorough understanding of the evolution of cognition and mechanisms underlying behaviors across many species. In addition to this, we believe that the theory and knowledge gained from the study of animal cognition will play an increasingly important role in helping us deal with the global challenges we face over the next 25 years and beyond. This includes conservation, maintenance of ecosystem processes and the global food threat. In this review, we consider the role that animal cognition has in a key ecosystem service, biological pest control.

As the human population grows, escalating demands for food have led to an increase in agriculture which is now the leading cause of habitat loss globally (Rudel et al. 2009). A result of this expansion, intensive farming methods have resulted in a decrease in beneficial crop pest predators, eventually increasing pest outbreaks and crop loss (Kitzes et al. 2008). Phytosanitary agents like pesticides and herbicides have long been the main solution to this issue (Carson 1962), however, their detrimental impacts on the ecosystem (Gonthier et al. 2014), crop health, and human well-being are now clear. Therefore, it is essential to mitigate crop loss while maintaining sustainable agriculture (Matteson 2000; Shukla et al. 2019). Global crop loss due to the combined effect of weeds, pests, and diseases is between 27 and 40% (Oerke and Dehne 2004), however, it is estimated that this increases to 48–83% without crop protection (Oerke 2006). Across all vegetation types, foliage, sap, and root-feeding pests cause major losses in crops (Agrawal 2011).

Modern approaches in biocontrol are directed towards increasing the abundance of natural enemies, this includes their importation, augmentation (mass rearing and release into fields), and conservation (Landis 2000). However, our knowledge of the efficiency of natural enemies is still extremely limited. This has led to failed efforts such as introducing Cane toads (*Rhinella marina*) as a predator for cane beetles. The cane toads have been shown to feed more on beneficial natural enemies than on crop pests, as well as being a threat to the native reptiles and mammals (Shuman-Goodier et al. 2019). To truly understand the factors that lead to predator success, it is essential to understand the crucial factors which determine how animals remember and evaluate food sources—the cognitive processes underlying their behavior. Here, we probe the present status of biocontrol and attempt to build case for the essential role of animal cognition within this framework.

## Biological pest regulation: the current approach

Biological pest regulation is a key component of Integrated Pest Management (IPM) in agriculture and has been used increasingly over the last two decades (Barratt et al. 2018). However, given the global population rise and increasing agricultural demands, the need for biological pest control in the twenty-first century is greater than ever before (Bale et al. 2008). The most commonly used organisms in natural pest regulation are invertebrate predators, parasites/parasitoids, microorganisms, and herbivorous arthropods (Garcia et al. 2020). Use of commercially reared natural enemies to regulate pests has been an age old practice. Approximately, 230 species of arthropod biological pest regulators are commercially available (van Lenteren 2012) and with more research on the efficiency of pest regulation by natural enemies, the number of pest predator species is likely to increase. An advantage of using insect biocontrol agents is their narrow range of specificity, their short life cycle, and the fact that their populations fluctuate in response to available pest density. Surprisingly, little attention has been given to potential vertebrate bioregulators. For example, 50% of bird species are predominantly insectivorous (Wenny et al. 2011; Sekercioglu et al. 2016). Nyffeler et al. (2018) estimated that insectivorous birds consume 400–500 million tons of arthropod prey globally per year, with approximately 28 million tons (~ 7%) coming from agricultural lands. Further, birds like falcons and owls are known to control vertebrate pests that include rodents (Whelan et al. 2015) and birds (Shave et al. 2018).

Though biocontrol is the ultimate choice for sustainable agriculture, as it currently stands, it has its limitations. Macfadyen et al. (2019) showed that most studies related to crop pests probed ecological and population aspects while studies on natural enemies are focused on studying their biology and ecology. From the perspective of the natural enemy, food varies in nutritional quality, quantity, and distribution, across seasons and between generations. Given this variation, behavioral adaptation is crucial in these changing environments, and learning provides a mechanism to cope with this uncertainty (Papa et al. 1993). Studying basic learning, cognition, and behavioral plasticity in pest predators, therefore, offers the potential to improve the performance of predators. To date, many strategies have been implemented to improve pest control but Integrated Pest Management strategies must evolve to remain effective. The idea of manipulating the behavior of the natural enemy is likely to have a substantial impact on the success of IPM strategies (Rodriguez-Saona et al. 2012). Making use of an animal’s learning and cognition represents a shift in approach and has the potential to a) improve our understanding of the performance of pest controllers and b) use this information to adapt their performance and improve service provisioning.

## Biocontrol through manipulation of pest cognition and behavior

Learning can be used to manipulate crop pest behavior, disrupting their impact on crops. There is evidence that aversive conditioning to semiochemicals can directly reduce infestation and increase preference for non-crop plants (Cunningham et al. 1998). However, the effect of this varies across the diverse life history phases in insects, and it seems to be more effective when experienced in the early stages for newly emerging pests (Westwick and Rittschof 2021). Volatile cues have also been used to modify oviposition sites (Bruce et al. 2005; Carlsson et al. 1999). For example, following a feeding association with a repellent and food in the last instar larval stage, adult female Cabbage looper moths (*Trichoplusia ni*) oviposit in plants that are blanketed by the repellent (natural latex) (Shikino and Isman 2009). Other caterpillars can learn to associate a plant's volatile cues with attractive and aversive gustatory stimuli. This can be used to alter their preference for a particular host plant (Salloum et al. 2011). Further, necromones (chemicals arising from dead individuals also known as death-recognition chemicals) can also serve as important stimuli for facilitating avoidance learning in pests and can be harnessed for biocontrol. There is substantial evidence that pests can learn through association. For example, adult female Silverleaf whiteflies (*Bemisia tabaci*) learn to avoid ovipositing on plants harboring predatory mites (Nomikou et al. 2003), whilst crickets (*Gryllus bimaculatus*) and desert locusts (*Schistocerca gregaria*) can learn to associate an odor with food rewards (Simões et al. 2011). In locusts, a single learning trial elicited bias for an odor for 4 h and multiple exposures helped to retain memory for 24 h (Simões et al. 2011). The fact that these pests are readily able to form associations between olfactory cues and food reward suggests that this approach could be highly effective in manipulating their decisions and redirecting their foraging away from crop plants. This is the main goal of biological pest regulation.

Alarm pheromones, which are characteristic of social or gregarious insects, also occur in important insect pests like Aphididae (aphids) and Thripidae (thrips). Flooding crop patches intermittently with alarm pheromones, aversive odors or necromones could be used to disperse groups of insects. Similarly, blanketing weeds or non-crop plants with aggregation pheromone can divert the pests to infest the non-crops from where they can be disposed or eradicated using sticky traps or bio-repellents. Trail pheromones in social insects and host marking pheromones in parasitoids can also be used to alter host preferences to non-crop plants. Flooding 35-acre cotton field plots with sex attractant pheromones of beet army-worms (*Spodoptera exigua*) (a serious pest of cotton) disrupted mating behavior for a substantial period (Shorey and McKelvey 1977). With such a pervasion of female scent, the males could not find females for more than 100 days, making the pest population less viable. The main idea behind utilizing cognition in pests is to enable them to learn that crop plants are unsuitable hosts. Combining the interventions which alter their preference with others that disrupt their mating can serve to reduce their population with every subsequent generation.

## Cognition in invertebrate pest controllers

In contrast to altering food preferences in crop pests to enhance biocontrol, cognition in natural pest predators could be used to increase prey specificity. Prey specificity in natural enemies (pest predators) is context-specific (Brodeur 2012). This has been used to understand the decline of populations or species (Howarth 1991; Simberloff and Stiling 1996), however, there has been no attempt to improve performance or manipulate behavior towards specific pest types (Brodeur 2012; Little et al. 2019). Influencing prey specificity presents scope to adapt predator behavior to make predators more efficient in their service provisioning.

Use of semiochemicals (organic compounds used by insects to convey specific chemical messages that modify behavior or physiology) can modify prey searching behavior and is also effective over longer distances (El-Shafie and Faleiro 2017). Aggregation pheromones from Coleopteran (beetle) and Hemipteran (true bug) predators are used to regulate Lepidopteran (butterfly and moth) pests (e.g. Sharma et al. 2019). Histerid (clown beetle) predators can locate their prey’s breeding sites in response to aggregation pheromone of the prey (van Lenteren et al. 2006), thus increasing the presence of this pheromone would attract more predators (though there is a risk that this might impact localization). Parasitic wasps are a large group of Hymenopteran biocontrol agents. Semiochemicals have been used to make parasitoids (insects whose larvae develop within another insect host) switch hosts to target invasive pest species. This is done by masking plants with host species-specific pheromones or with chemicals that the plant releases in response to pests (Barratt and Johnstone 2001). For example, when a newly emerged Red Scale Parasite wasp (*Aphytis melinus*) is exposed to kairomones (semiochemicals that are beneficial to organisms that detect the cues) of the California red scale insect (*Aonidiella aurantii*; a pest of citrus), it increases the preference and efficiency of red scale parasite wasp for locating this prey (Hare et al. 1997). Aphid parasitoid wasps (*Aphidius rhopalosiphi*), can even differentiate between the different cultivated varieties of wheat (Wickremasinghe and van Emden 1992). Interestingly, adult learning is also reported in another aphid parasite wasp (*Aphidius colemani*). Upon emergence from their own mummies when the aphid parasitoid wasps were given an oviposition experience with an aphid from the other plant, they showed a preference for the plant on which this aphid had been reared rather than the plant on which they themselves had developed (Storeck et al. 2000), allowing the potential to manipulate prey choice.

Visual cognition is another essential aspect of biocontrol. Endoparasitoid wasps (*Venturia canescens*) prefer yellow, which is the most abundant color among natural flowers in temperate regions (Lucchetta et al. 2008). Lepidopteran eggs vary in color from white, yellow, or green, to bright orange or red, and are often laid in a multicolored clutch or changing color over time (du Merle and Brunet 1991). This provides an immediate or short-range visual cue that parasitic wasps can use to identify the location and viability of eggs.. *Trichogramma* sp. (endoparasitic wasp) show a preference for yellow and white color eggs over black as the latter signify either parasitized or otherwise damaged eggs (Lobdell et al. 2005). This natural preference could be harnessed by exposing invertebrate bioregulators with these (or other) color preferences to appropriate eggs of the target crop pest. In addition, their ability to discriminate colors could be harnessed to build an association between color and a target pest species.

The above summary indicates the flexibility in pest predators’ behavior which can be harnessed as a strategy to improve the effectiveness of augmentative release programs to control specific crop pests. (Vet et al. 1995). Considering its rapid response and flexibility, inducing a chemical response is feasible even in crop fields without augmentation. Spraying target crop patches with kairomones can help to aggregate the parasitoids in sites where pest infestation is high. However, since the response can be expected to vary across life history phases, unless the exact intensity of response is known multiple interventions encompassing the whole life cycle of an insect predator will be required.

## Cognition in vertebrate pest controllers

Though insects are sensitive to a variety of cues including spatial, visual, olfactory, and tactile (Little et al. 2019) they interact with the environment and ecosystem in their niche, the spatial scale of which are different from vertebrates (Lunau 2014). In addition, vertebrates may offer greater flexibility and potential for long-term change.

Insect crop pests show a range of salience in their color, structure, size, chemical cues, odor, movement, occurrence, and behavior. This makes them difficult targets for pest management which often requires a combination of strategies. However, this very diversity offers a wide array of opportunities to condition predators.

## Taste discrimination

Taste (Gill et al. 1998) can be an excellent tool to enhance avoidance learning. Animals rapidly learn salient features e.g. coloration associated with taste (Gamberale-Stille 2001). The bitter taste of grasshoppers dyed in specific colors elicited avoidance in Swinhoe's lizard, (*Diploderma swinhonis)* this discrimination was remembered for 60 days (Ko et al. 2020). By retaining information from the previous encounter (Bracis et al. 2015), animals can avoid re-assessing the value of food (Armstrong et al. 2012). Aversion to a bitter taste helps animals to avoid ingestion of toxic secondary plant compounds accumulated in potential prey. Some insects carry toxins that taste bitter (Pasteels et al. 1983). Hymenopteran pests (for example ants) have accessory glands in the female reproductive system that have become modified to produce toxic proteins. However, aposematic prey is not always unprofitable. Some studies speculate that prey may also contain valuable nutrients along with toxins that make them appropriate potential food (Barnett et al. 2007). Bitterness varies with the concentration of toxins (Schafer et al. 1983), and animals have an inherent tolerance for certain levels of toxins (Skelhorn and Rowe 2007). Predators therefore face a trade-off between the cost of ingesting a toxin to gain valuable macro and micronutrients (Barnett et al. 2007). Avian predators are able to taste and discriminate between defended prey based on their level of defense (Skelhorn and Rowe 2006). For example, blue tits (*Cyanistes caeruleus*) modify their foraging strategy not just to avoid toxins but to maximize nutrient intake in a tradeoff with minimal tolerable toxin consumption (Skelhorn and Rowe 2010). Birds can also quantify different levels of prey defense chemicals and discriminate between visually identical prey on the level of their chemical investment (Skelhorn and Rowe 2006). Reptiles can selectively choose palatable and sweeter prey while avoiding distasteful or bitter prey items (Stanger-Hall et al. 2001; Shanbhag et al. 2010). Taste aversion by itself or in association with cue manipulation (e.g. color – see below) can result in effective, rapid and long-term learning where an animal’s prey preference can be manipulated. Thus manipulating levels of unpalatableness in non-pest prey can be used to encourage predators to forage on specific crop pests, thereby reducing pest abundance. Alongside using taste aversion to shift prey preference, there is a possibility that predators can be trained to discriminate between various bitterness levels in crop pests. This can provide scope to regulate toxic pests that might show resistance to other measures or avoided by other natural enemies.

## Visual cues

Animals’ innate color preferences evolve based on the colors they encounter most in their environment or those which contrast most with the background (Lunau et al. 2018). Discriminating between color or color preference is a simple way for animals to find and select appropriate food. However, color preference appears to be context specific. For example, domestic chicks and blackcaps (*Sylvia atricapilla*) prefer green insects over red ones but do not show color preferences when presented with green and red fruits (Gamberale-Stille et al. 2007). Blue tits (*Cyanistes caeruleus*) and great tits (*Parus major*) prefer red color in a positive context, but this is also impacted by an individual’s age and previous experience. Juveniles of both species preferred green almonds regardless of their prior experience, whereas adults chose red before green only after having a positive experience with the reward (Teichmann et al. 2020). As such, it is essential to understand the role that preferences have in pest control and, as these can be manipulated by experience, there is potential for manipulation to ensure predators target pest species.

There is also evidence of learned color discrimination across the animal kingdom (Fish- Escobar-Camacho 2019; reptiles- Soldati et al. 2017; birds- Teichmann et al. 2020; Pene et al. 2020). For example, Cichlids use color to detect, identify and discriminate different foods, offspring (if they are mouth-brooders), and mates (Escobar-Camacho and Carleton 2015; Price et al. 2008). Such information can be utilized for designing bioregulation programs. For example, training predators to associate specific colors with positive or negative outcomes can improve the efficiency of control. Alongside this, other visual factors such as luminance, size, pattern, and contrast may also be likely candidates for this learning.

## Odor cues

Olfaction is an important sense in many species (Roper and Marples 1997). Birds have been documented to use olfactory cues in locating and discriminating between foods (Roper 1999). Reptiles like Varanids (Monitor lizards) are known to detect carrion from a distance of 11 km (Auffenberg 1981). Sleepy lizards (*Tiliqua rugosa*) can detect smoke via olfaction (Mendyk et al. 2020). Insectivorous lizards, five-lined skink (*Eumeces fasciatus)* are known to discriminate chemicals from prey while omnivorous lizards respond to both plant and animal prey chemicals *(Elaeophora schneideri, Scincus mitranus)* (Cooper et al. 2000)*.*

Ability to discriminate between multiple prey odor can be of advantage to identify and locate prey. For example, Sand goannas (*Varanus gouldii*) can differentiate between odors of crickets, mice and geckos (Garrett and Card 1993).

Crop pests are defended by specific odor (e.g. Stink bugs) and olfaction in pest predators can a valuable technique by which prey specificity can be manipulated in natural predators. However, such intervention will require further investigation.

## Auditory cues

Most of the Orthopteran insects like grasshoppers, locusts and crickets are phytophagous, i.e., they feed on plant parts and are major defoliator pests (Ingrisch and Rentz 2009). This is the group of arthropods that exhibit vibrational and acoustic signaling. Most lizards have good auditory sensitivity across the range from 100 to 10,000 Hz (Capshaw et al. 2021). Reptiles also show evidence of associating sounds with foraging decisions. Kalahari tree skinks (*Trachylepis spilogaster*) exploit weaver bird (*Philetairus socius*) alarm calls when foraging (Lowney et al. 2020). Weaver birds emit alarm calls in response to the presence of African pygmy falcons (*Polihierax semitorquatus*), which also predates on the skinks. Skinks are able to learn to associate the sound of weaver bird alarm calls with predatory threat when they forage in riskier habitats and use it as an early warning signal to flee. However, not enough evidence exists regarding tonal quality discrimination ability. Some studies suggest lizards learn to locate food by detecting mechanical vibration (Hetherington 1989). Vibrational cues and acoustics can therefore be used as a potential strategy to indicate the presence of pests to predators, thus promoting their role in regulation.

## Numerosity and quantity judgment

Numerical processing or quantity judgment is considered adaptive as it allows animals to select, for example, a larger number of social companions (Bisazza et al. 2010) or a larger quantity of food (Uller et al. 2003). In its simplest form, it represents the ability to differentiate between “more” or “less” (Reznikova 2007; Stancher et al. 2015), which helps to maximize the food items found in one place in a single visit (Stancher et al. 2015). There is even evidence that animals can perform numerical discriminations (fish- Piffer et al. 2013; Lucon-Xiccato et al. 2015; frog, Stancher et al. 2015; reptile- Szabo et al. 2021; birds, Corliss et al. 2021; mammals- Ward and Smuts 2007).

Food availability is a strong motivator to revisit a food patch and animals can learn to associate cues with different quantities and qualities of food (Soldati et al 2017). These skills can be harnessed to improve predator foraging efficiency; artificial cues could be used to provide predators with information about the presence of large quantities of pests or key food patches to visit. For example, grasshoppers generate swarms which result in substantial loss of food crops (Naskrecki 2001); in these instances cues can be added that support numerical discrimination of the predators, allowing them to rapidly adapt their foraging and selectively forage on patches with large numbers of pests.

## Social learning

Individual learning is costly as it is time-consuming, energy-demanding, and increases risks of predation (Hoppitt and Laland 2013). A shortcut to learning new information is to observe the behavior of a conspecific (Wilkinson et al. 2010). Guppies in the wild can socially learn foraging locations (Reader et al. 2003); and this can alter the distribution of individuals over resources (Beauchamp et al. 1997). As a result, discovered food patches are likely to be exploited more rapidly, but the exploitation of initially overlooked patches will be slowed. There is evidence that animals can learn about feeding locations (Midford et al. 2000), food items (Clark 2010), hunting strategies (Kitowski 2009), handling and feeding techniques (Boogert et al. 2008), accessing otherwise inaccessible goals (Wilkinson et al. 2010) and even tool use (Auersperg et al. 2014) from observing other individuals. Some species are also able to learn from heterospecifics (Brodin and Urhan 2014). There is also an instance of socially mediated food avoidance in birds as well (Sherwin et al. 2002).

Social learning could therefore be used to influence predator foraging both in terms of the position of food but also prey specificity. As such, the use of conspecifics, or fake conspecifics (be they robots or non-moving artificial stimuli; see Frohnwieser et al. 2017) can be used to enhance pest control performance.

## From theory to practice: a potential approach

Oriental garden lizards (*Calotes versicolor*) are important pest controllers across South East Asia. They are generalist insectivores that are found abundantly in agricultural land. They are highly adaptable and are also prolific breeders (Ghosh and Basu 2020). As such they are an ideal model species for testing these concepts. There is evidence of rapid color discrimination and taste aversion learning in this species, with one day olds successfully associating a color with a taste after only a single exposure (Shanbhag et al. 2010). Further, adults are able to rapidly discriminate between non-conspicuous colors (under preparation, Ghosh and Wilkinson). Most crop pests are cryptic in nature and evidence of such learning provide an opportunity to manipulate biocontrol. Given that taste aversion learning is so rapid, and also long-lasting, such an intervention is inexpensive and can be done directly in the field. Thus, a taste aversion paradigm could be used to reduce the likelihood of this species predating upon non-pests. We would anticipate that this would increase the prey specificity towards targeting a major pest by shifting prey choice.

Garden lizards respond to the sound of crickets and are able to learn to differentiate between foraging patches based on prey density (Ghosh et al. unpublished data). However, crickets tend to sing at times that garden lizards are less active (to avoid predation). This preference can be harnessed by playing recordings of crickets singing at infestation sites at times when garden lizards are most active. We would anticipate that this would result in a suppression of crop pests.

Since these lizards are ambush predators and territorial we could assess foraging behavior of individuals using a mark and recapture study in the field. By collecting fecal samples we could explore the specificity of their diet and assess the duration of the effect of the manipulation. Consequently, the frequency at which we need to manipulate the taste can be decided accordingly.

## Summary

Most pest management strategies are ecologically based and are practiced in isolation and therefore the impact is limited. Here, we introduce the idea that our understanding of the cognition of both pests and their predators should be integrated into ecologically based pest management strategies. This requires further pure work in the field of animal cognition in combination with key applied work examining the direct impact of cognition and cognitive interventions on pest control. This new approach will not only reduce agricultural loss but also has the potential to increase the diversity of many predator species which have declined as the result of agricultural intensification (Reading et al. 2010; Ghosh and Basu 2020).

## Data Availability

This study has not generated any data as it is a review.
